# CONSTRICTOR: Constraint Modification Provides Insight into Design of Biochemical Networks

**DOI:** 10.1371/journal.pone.0113820

**Published:** 2014-11-25

**Authors:** Keesha E. Erickson, Ryan T. Gill, Anushree Chatterjee

**Affiliations:** 1 Department of Chemical and Biological Engineering, University of Colorado, Boulder, Colorado, United States of America; 2 BioFrontiers Institute, University of Colorado, Boulder, Colorado, United States of America; University of Houston, United States of America

## Abstract

Advances in computational methods that allow for exploration of the combinatorial mutation space are needed to realize the potential of synthetic biology based strain engineering efforts. Here, we present Constrictor, a computational framework that uses flux balance analysis (FBA) to analyze inhibitory effects of genetic mutations on the performance of biochemical networks. Constrictor identifies engineering interventions by classifying the reactions in the metabolic model depending on the extent to which their flux must be decreased to achieve the overproduction target. The optimal inhibition of various reaction pathways is determined by restricting the flux through targeted reactions below the steady state levels of a baseline strain. Constrictor generates unique *in silico* strains, each representing an “expression state”, or a combination of gene expression levels required to achieve the overproduction target. The Constrictor framework is demonstrated by studying overproduction of ethylene in *Escherichia coli* network models iAF1260 and iJO1366 through the addition of the heterologous ethylene-forming enzyme from *Pseudomonas syringae*. Targeting individual reactions as well as combinations of reactions reveals *in silico* mutants that are predicted to have as high as 25% greater theoretical ethylene yields than the baseline strain during simulated exponential growth. Altering the degree of restriction reveals a large distribution of ethylene yields, while analysis of the expression states that return lower yields provides insight into system bottlenecks. Finally, we demonstrate the ability of Constrictor to scan networks and provide targets for a range of possible products. Constrictor is an adaptable technique that can be used to generate and analyze disparate populations of *in silico* mutants, select gene expression levels and provide non-intuitive strategies for metabolic engineering.

## Introduction

Developing better predictive models of metabolic network behavior is essential for exploiting microbial cell factories for biochemical production. Though microbial cell factories have been successfully engineered for the overproduction of a number of products including ethanol [1], [2], isobutanol [3], [4], alkanes [5], [6], and thermoplastics [7], [8], continued advancement in strain engineering requires increased understanding of the impacts of combinatorial mutations on overall physiology [9]–[12]. Recent high-throughput genome engineering strategies such as trackable recursive multiplex recombineering (TRMR) [13] and multiplex automated genome engineering (MAGE) [14] have allowed for rapid genome-wide search and combinatorial manipulation of microbial chromosomes [15]. Computational design tools that allow for exploration of the combinatorial mutation space are thus valuable tools to guide such genome-wide experimental techniques, providing an avenue for analyzing the system-wide effects of genetic manipulations.

Biological networks have been described using kinetic models for decades [16]–[21]; however, these require kinetic parameters and are computationally more complex. Steady state models based on stoichiometric and flux constraints provide a comparatively straightforward platform for analysis of complex systems where kinetic parameters are not required. Among such steady state modeling techniques, FBA is one of the most prevalent for modeling cellular metabolism. FBA has been applied to central metabolism-focused networks containing a few hundred reactions [22], [23] as well as genome-scale reconstructions containing thousands of reactions, and has been demonstrated to successfully predict growth [24], [25], estimate theoretical yields from heterologous pathways [26], locate engineering targets to maximize product yield [27], and design antibiotics [28]. While typical FBA is limited in that kinetics, thermodynamics, and gene-regulation are often neglected, FBA has remained in favor because it provides a straightforward platform for analysis of complex systems.

A number of studies have been published that use the FBA framework to reveal non-obvious network characteristics. Optknock was developed for designing *in silico* knockout strains that redirect flux from biomass to biochemical production [29]. OptReg and OptForce expanded upon the Optknock framework by exploring combinations of gene up-regulation, down-regulation, and deletion to find metabolic engineering targets [30], [31]. Because combinatorial analysis of gene targets with FBA scales exponentially, alternate algorithms have been applied to efficiently study metabolic networks. Genetic algorithms [32], successive linear programming [33], and local search [34] have succeeded in rapidly locating target modifications with order of magnitude reductions in computational time. These approaches can incorporate mixed integer linear programming problems, such that simulated constraints generated via OptKnock or others can be solved with efficient algorithms; examples include the use of OptKnock and OptGene [35] and OptReg*LS [33]. The current FBA-inspired methods allow optimization of fluxes and identification of gene targets for deletion, up-regulation, and down-regulation; however, there continues to be a need for alternate approaches that allow for comparison between subtle variations in gene expression and metabolic network behavior.

**Figure 1:**
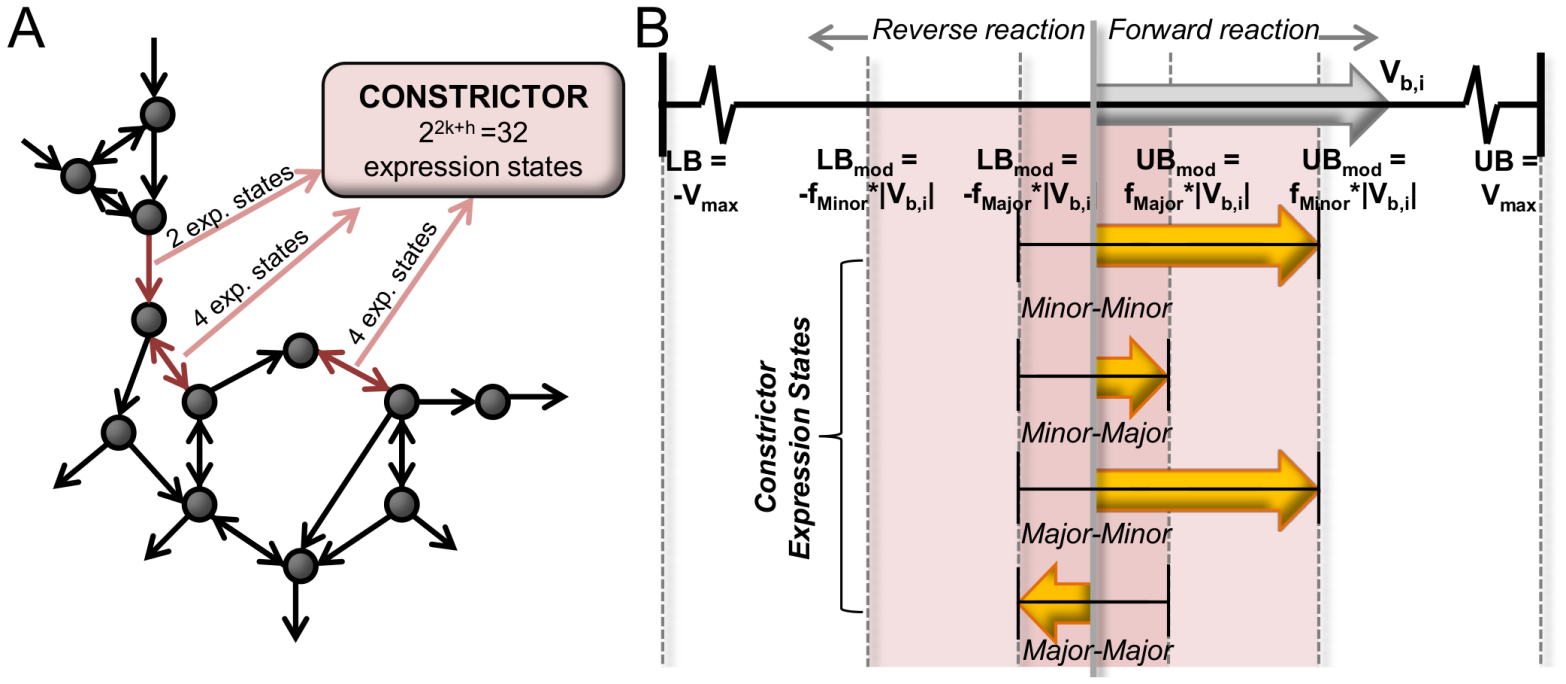
Overview of the Constrictor framework. (**A**) Constrictor explores the impact of various “expression states” corresponding to different levels of expression of metabolic enzymes in the reaction network to obtain *in silico* mutants with diverse properties. When multiple reactions are targeted simultaneously, each combination of expression states is optimized, such that the total number of expression states becomes equal to *2^2k+h^*, where *k* and *h* are the number of reversible and irreversible reactions respectively that are targeted by Constrictor. (**B**) Constrictor algorithm: Constrictor uses FBA to combinatorially explore the impact of modifying lower and upper bounds of enzyme associated reaction fluxes in a metabolic pathway. FBA is first run with lower (LB) and upper bounds (UB) set to -v_max_ and v_max_ to obtain a baseline flux solution. The baseline solution for a single reaction to be targeted by Constrictor is shown in the uppermost arrow, labeled v_b_(t_i_). Constrictor is used to generate expression states based on percentage reductions of the flux bounds from the baseline flux. When considering two possible adjustments: a “Minor” restriction and a “Major” restriction, modified bounds (LB_mod_ and UB_mod_) are calculated as shown. For a reversible reaction four expression states exist which correspond to the degree to which the lower and upper bounds are restricted (“Minor-Minor”, “Minor-major”, “Major-minor” and “Major-major”). For irreversible reactions, two expression states exist (“Major” and “Minor”).

In this report, we introduce Constrictor, an FBA strategy to analyze the impact of various degrees of inhibitory effects of genetic mutations observed during genome engineering experiments on the performance of biochemical networks. This is achieved by restricting flux bounds by “Minor” and “Major” levels below the steady state level in order to simulate different extents of inhibition caused by mutations. Constrictor generates unique *in silico* strains, each representing an “expression state”, or a combination of gene expression levels (Fig. 1A-B). Sets of expression states are based on percentage reductions in flux, such that the impact of finely tuned gene repression can be studied rationally. Constrictor is employed to investigate heterologous ethylene production. Ethylene is an important intermediate compound that can be converted into a multitude of products, including plastics like polyethylene, polyethylene terephthalate, and polyvinylchloride, as well as fuels like gasoline, diesel, and ethanol [36]. Biological synthesis of ethylene is a promising avenue to reduce fossil fuel requirements and industrial emissions associated with chemical manufacturing.

Ethylene biosynthesis utilizing the ethylene forming enzyme (EFE) from *Pseudomonas syringae* has been studied in a number of organisms, including *Saccharomyces cerevisiae* [37], *Synechococcus elongatus* [38], *Synechocystis* [36], [39], *Pseudomonas putida* [40], and *E. coli* [39], [41], [42]. The ethylene forming enzyme from *P. syringae* catalyzes a reaction between L-arginine, 2-oxoglutarate, and oxygen to form ethylene, carbon dioxide, succinate, guanidine, and (S)-1-pyrroline-5-carboxylate (P5C) [43]. In yeast, the highest ethylene yield is reported to be 0.01 mol ethylene/mol glucose, which is approximately 1% of the theoretical maximum yield, reported as ranging between 0.73 and 0.87 mol ethylene/mol glucose [26]. Engineered *P. putida* strains have been shown to produce 0.14 mol ethylene/mol glucose at a production rate of 2.86 mmol/gdcw/hr, which is the highest reported yield and production rate for ethylene in any microbial host [40], [44]. In *E. coli*, the maximum reported ethylene production rate is 0.008 mmol/gdcw/hr, which is less than 1% of the production rate in *P. putida* [45]. We choose *Escherichia coli* as the ethylene production host for the model, as *E. coli* represents a common bacterial production strain with a simple metabolic network, and low ethylene production in *E. coli* indicates the potential for optimization. Here, modifications to *E. coli* metabolism are combinatorially analyzed using Constrictor, with the goal of revealing gene repressions that can inform experimental efforts and improve bioethylene yields as well as yields of alternate products.

## Methods

### Metabolic models for ethylene production

To study ethylene production in *E. coli*, the *E. coli* core model iAF1260 was modified to build a focused metabolic network for heterologous ethylene production. The core model contains 95 reactions and 72 metabolite species, derived from the iAF1260 genome scale reconstruction (http://gcrg.ucsd.edu/Downloads/EcoliCore) [46]. Ethylene production was modeled according to the *Ps. syringae* EFE reaction [43]. The BIGG (http://bigg.ucsd.edu/), KEGG (http://www.genome.jp/kegg/), and Ecocyc databases (http://ecocyc.org/) were used to locate the additional reactions and metabolites needed to complete the model [47]–[49]. Complete lists of the 114 reactions and 89 metabolites in the core ethylene producing model are available in the Supplement (Tables S2-S3 in File S1 and Fig. S1). Out of the 114 reactions in the core metabolic model, 66 reactions are reversible, and 48 reactions are irreversible. Unless otherwise stated, presented results are derived from the ethylene core model. For genome scale studies, the EFE reaction was added to the *E. coli* iAF1260 and the iJO1366 models [46], [50]. Modifications and constraints applied to the core and genome scale models are described in the following sections and summarized in Supplementary Information (Table S1 in File S1). MATLAB (The Mathworks, Inc., Natick, MA) was used with the Gurobi Optimizer (Gurobi Optimization, http://www.gurobi.com) to execute each FBA linear programming problem. All models were modified and solved using the COBRA toolbox [51]. Constrictor software and SBML models created for this report are provided at http://www.colorado.edu/UCB/chatterjeelab/.

### Conditions and parameters for ethylene production models

Aerobic conditions are considered for all studies by setting a maximum glucose uptake of 10.5 mmol per gram dry cell weight per hour (mmol/gdcw/hr) and a maximum oxygen uptake of 15 mmol/gdcw/hr [25] (Equation 1). The lower bound on the ATP maintenance reaction is held at a constant value of 3.15 mmol/gdcw/hr [50]. Exponential growth is simulated by enforcing a minimum bound on the biomass reaction [29], [33]. The required growth rate for this study is 0.3 hr^-1^, defined according to experimental observations for *E. coli* MG1655 in minimal media (Fig. S2). Results for other minimum growth rates are summarized in Fig. S3. Ethylene yield is defined as the absolute value of the ratio of flux through the ethylene demand reaction and the glucose uptake reaction (mol ethylene/mol glucose). Ethylene production rate is defined as the flux value through the ethylene demand reaction (mmol/gdcw/hr).

### Constrictor framework: Exploring impact of constraints on lower and upper bounds for all fluxes in a network

Constrictor explores the system response for combinations of “inhibitory” rate adjustments (Fig. 1A-B). First, the FBA linear programming problem is solved to obtain the maximum theoretical ethylene yield (Equation 1): 
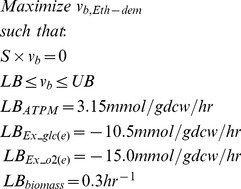
(1)


Where the vector *v_b_* represents the baseline flux through each of *r* reactions and *S* is the stoichiometric matrix for the reaction network. For the majority of results presented, we maximize ethylene production in order to study maximum theoretical ethylene yield, while enforcing a minimum growth rate in order to simulate metabolically relevant flux distributions. To demonstrate that alternate objectives are supported, we also describe a study that maximizes growth (via biomass reactions). The *LB* and *UB* vectors contain the lower and upper bounds of the flux for each reaction, respectively, and limits are placed on ATP maintenance, glucose uptake, and oxygen uptake (Equation 1). Other reactions are allowed to vary between -v_max_ to v_max_ mmol/gdcw/hr for reversible reactions, and 0 to v_max_ mmol/gdcw/hr for irreversible reactions. We set v_max_ to 1000 mmol/gdcw/hr indicating unlimited flux through each reaction of the metabolic network. Steady state conditions are assumed, such that the rate of metabolite change in the system is equal to zero.

A representative solution (*v_b_*, of length *r*) to this linear programming problem is determined for the baseline strain without any genetic modifications. Though alternate optimal solutions exist with equivalent maximized ethylene production [52], in this case as in others it is not necessary to determine the range of flux variability [23], [26], [33], [53], here because the intention is to generate derivative flux distributions corresponding to modifications of target reactions. Thus, we select one representative “baseline” solution as the starting point on which the Constrictor algorithm is subsequently applied. Constrictor targets metabolic reactions by constricting the lower and upper bounds of flux through the targeted reaction(s) at “Minor” and “Major” levels (Fig. 1B). Scalar “restriction factors” *f_Minor_* and *f_Major_* are coefficients that are used to modulate the upper and lower bounds of the targeted metabolic reaction(s) at Minor and Major levels (Equation 2). The restriction factors are user defined, range between 0 and 1, and represent percentage reductions in flux. Unless otherwise specified, the restriction factors *f_Minor_* and *f_Major_* are set to 0.8 and 0.2, respectively. For a set of ‘*n*’ metabolic reactions targeted by Constrictor (input into the vector *t*), all possible combinations of 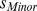 and 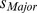 are generated (Equation 2). Each targeted reaction is multiplied by the baseline flux value to define specific expression values (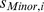, 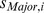), where *i* = 1 through *n*. 
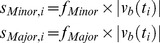
(2)


The modified lower and upper bounds of the flux (*LB_mod_* and *UB_mod_*) for each reaction respectively are obtained by replacing the original lower and upper bounds used in the baseline solution by the specific expression values (Equation 3). By using this approach, reaction flux in either direction of reversible reactions is constrained according to major and minor reductions of the magnitude of baseline (Fig. 1B). This allows flux to switch directions if subsequent FBA runs find this to be optimal. As a result for a reversible reaction, there exist 2^2^ possible combinations of adjusted flux bounds (“Minor-Minor”, “Minor-Major”, “Major-Minor” and “Major-Major”) (Equation 3). Similarly, for an irreversible reaction there exist 2^1^ possible combinations (“Major” and “Minor”). 
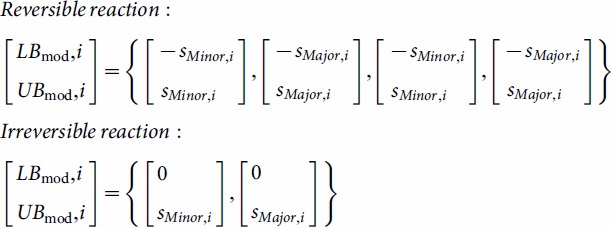
(3)


The number of combinations generated, herein referred to as “expression states”, is equal to *c* = *2^2k^×2^h^*, where *k* and *h* are the number of reversible and irreversible reactions respectively that are targeted by Constrictor (Fig. 1A).

Reactions that are inactive in the baseline solution (corresponding to zero flux through the reaction) are excluded from modifications, and allowed to vary between the original upper (v_max_) and lower bounds (-v_max_) for Constrictor FBA optimizations. The biomass, glucose uptake, oxygen uptake, and ATP maintenance reactions are also excluded from Constrictor modifications. The resultant FBA linear programming problem is solved to obtain the globally optimal solution for ethylene production for each of the *c* expression states, where each column (*j* = 1 through *c*) is iteratively selected as the flux boundaries (Equation 4).
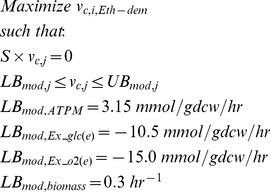
(4)


The final flux solution matrix, *v_c_*, contains *r* rows and *c* columns. Each column in *v_c_* represents the solution obtained from a unique expression state.

## Results

### Modification of individual metabolic reactions increases ethylene yield

**Figure 2:**
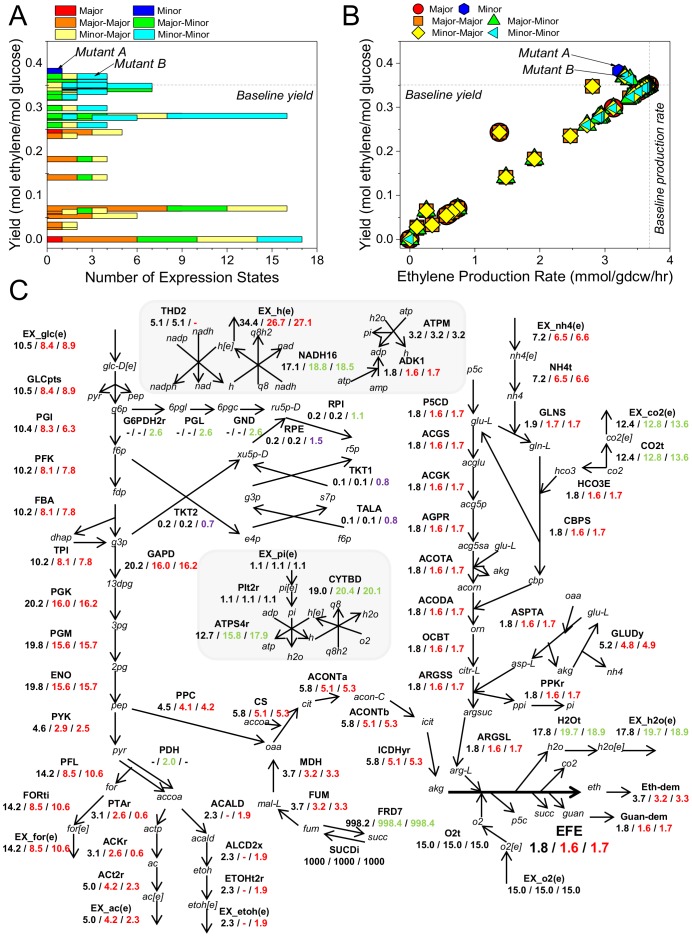
Constrictor Study on Individual Reactions. (**A**) The ethylene yields for each expression state (relative to baseline) are shown on the vertical axis, and the corresponding number of the expression states is shown on the horizontal axis. Expression states are indicated in the legend. “Major” and “Minor” represent modifications to the upper bound of irreversible reactions. Other expression states (e.g. “Major-Major”) describe the modifications to lower and upper bounds of reversible reactions, respectively. (**B**) The solutions obtained from targeting each active reaction are plotted according to their yield and production rate. Horizontal and vertical lines indicate baseline yield and baseline production rate, respectively. (**C**) Metabolic pathway map comparing flux of metabolic reactions (mmol/gdcw/hr) between the baseline case and mutants A and B (baseline/mutant A/mutant B). Red, green and black text indicates that the magnitude of flux in the mutant is lower, higher, and equal to the baseline flux, respectively. Purple text indicates that the direction of the reaction is opposite to the baseline case.

The baseline FBA solution in the core model with aerobic conditions predicts an ethylene yield of 0.35 mol ethylene/mol glucose. Reactions carrying non-zero flux in the baseline solution are referred to as “active reactions”, whereas as those with zero flux are termed “inactive reactions”. Out of the 114 reactions in the core model, 72 reactions were found to be active in the baseline case, including a flux of 0.3 hr^−1^ through the biomass reaction. The Constrictor framework was used to iteratively target each reaction in the core ethylene network, with combinations of restrictions to 20% and 80% of the baseline flux. Individual modification of each of the 66 reversible reactions and 48 irreversible reactions in the core metabolic network would generate 360 expression states or FBA studies, as there are four possible expression states for each reversible reaction and two expression states for every irreversible reaction. However, Constrictor does not target the 42 reactions found to be inactive in the baseline solution, since the optimization of reaction network with modifications to inactive reactions results in solutions identical to the baseline case. Applying Constrictor to the remaining 72 active reactions gives rise to 222 expression states. While 12 of these expression states converge to the baseline solution and another 196 expression states decrease ethylene yield, 14 expression states positively impact the ethylene yield with respect to the baseline solution (Fig. 2A-B). The expression states reveal a range (4–9%) of elevated ethylene yields when compared to baseline case (Fig. 2A). For majority of Constrictor modifications, restrictions to target reactions result in a proportional reduction in flux throughout the network, as demonstrated by the linear relationship between yield and production for the majority of expression states (Fig. 2B). However, certain expression states impact the network in a nonlinear fashion, causing disproportionate shifts in flux that can increase yield. These “off-diagonal” solutions highlight imbalances in the network, as described below for two high yield solutions.

The maximum yield of 0.38 mol ethylene/mol glucose, a 9% increase over the baseline yield is found for *in silico* **mutant A** when glucose transport (GLCpts) is held to 80% of the baseline value. In **mutant B**, yield increases by 6% (0.37 mol ethylene/mol glucose) with respect to baseline when either glyceraldehyde-3-phosphate dehydrogenase (GAPD) or phosphoglycerate kinase (PGK) is restricted to 80% of baseline (Table 1). The differences between the baseline and the mutant flux distributions are displayed in Fig. 2C (Table S4 in File S1). We observe that in the baseline case ethanol is produced, which indicates the presence of anaerobic limitations, despite the aerobic constraints simulated. In mutant A, the ethanol production pathway (ACALD, ALCD2x, ETOHt2r, and EX_etoh(e)) is completely off, while pyruvate dehydrogenase (PDH) activity increases. PDH reduces NAD^+^ to NADH, while ACALD and ALCD2x in the baseline solution regenerate NAD^+^ supply. The shifts in these reactions indicate that the ethanol production pathway is active in the baseline solution in order to balance NAD^+^/NADH. Constrictor targeting of GLCpts reduces flux through glycolysis, which reduces the amount of NAD^+^ converted to NADH via GAPD. Up-regulation of PDH flux (by 2 mmol/gdcw/hr with respect to baseline) is insufficient to compensate for the decrease in GAPD activity (by 4.2 mmol/gdcw/hr with respect to basline), so the ethanol production pathway shuts off and we observe increased flux through NADH dehydrogenase (NADH16). The decrease in ethanol production when the flux requirement for NAD^+^/NADH balance is reduced gives rise to improved ethylene yield in mutant A.

**Table 1:** Mutants identified through Constrictor modifications in the ethylene core network (f_minor_ = 0.8, f_major_ = 0.2).

Strain	Reactions Modified (Individually)	Expression State	Ethylene Yield	% ΔYield (with respect to baseline)	Growth Rate (hr^-1^)
Baseline	-	-	0.35	-	0.3
*Upper limit on glucose uptake*					
Mutant A	GLCpts	Minor (80% of baseline)	0.38	+9%	0.3
Mutant B	GAPD, PGK	Minor (80% of baseline)	0.37	+6%	0.3
Not viable	Ex_nh4(e)^†^, NH4t^†^, GLCpts^†^, RPI*, Ex_pi(e)*, PIt2r*	^†^Major (20% of baseline) *Either Major or Minor	0	−100%	0
*Fixed glucose uptake*					
Mutant C	GAPD, PGK	Minor	0.18	−48%	0.45
Mutant D	ENO, PGM	Minor	0.20	−43%	0.43
Not viable	ENO^†^, GAPD^†^, PFK^†^, PGK^†^, FBA^†^, PGM^†^, TPI^†^, Ex_nh4(e)^ †^, NH4t^†^, RPI*, Ex_pi(e)*, PIt2r*, GLCpts*	^†^Major *Either Major or Minor	0	−100%	0

Similarly, mutant B also has a lower ethanol yield than the baseline (albeit the decrease is only 2.5%), again due to reduced NADH production by GAPD. In mutant B, as opposed to mutant A, we note that limiting GAPD or PGK causes a portion of the upstream flux to divert through the pentose phosphate pathway (G6PDH2r, PGL, GND, RPE, RPI, TKT1, TALA, and TKT2). By this mechanism, some carbon dioxide is released via phosphogluconate dehydrogenase (GND), which reduces the amount of carbon that passes through GAPD or PGK and allows the Constrictor constraint to be satisfied. Additionally, the pentose phosphate pathway produces NADPH, which is used by glutamate dehydrogenase (GLUDy) and N-acetylglutamylphosphate reductase (AGPR) to synthesize precursors of L-arginine. We note that flux decreases through the NAD(P) transhydrogenase (THD2), presumably because the NADPH produced by G6PDHyr and GND is sufficient to meet the NADPH requirement of the L-arginine biosynthesis pathway. Increases in ATP synthase (ATPS4r), cytochrome oxidase (CYTBD), and NADH16 flux for both mutants A and B show that additional cofactors also influence ethylene production in a non-intuitive manner, likely related to oxygen deficiency. Certain expression states are not able to be solved, pointing to bottlenecks in the network. For instance, neither a 20% or 80% reduction in phosphate uptake or ribose-5-phosphate isomerase (RPI) activity is tolerated, nor is an 80% reduction in nitrogen or glucose uptake (Table 1).

Since decreasing glucose uptake improved yield in mutants A and B, we next examined the impact of fixing glucose uptake to 10.5 mmol/gdcw/hr during Constrictor analysis. Under this constraint none of the expression states lead to increased yield, though increased growth rates are noted when reactions in glycolysis are targeted (**mutant C** and **D**, Table 1 and Table S4 in File S1). Restricting glycolysis forces flux to divert through the pentose phosphate pathway, increasing production of biomass precursors. Again, restricting certain reactions including those in glycolysis as well as nitrogen, phosphate, and glucose transport (Table 1) is not viable due to stoichiometric violations. When we plot yield versus production rate for the solutions obtained with fixed glucose, no off-diagonal solutions are observed (data not shown). Thus, in order to further explore possible ethylene yields, we continue to allow for flexibility in substrate uptake, and enforce only maximum uptake rates and a minimum growth rate.

### Constrictor allows for expression state tuning to locate higher yields

**Figure 3:**
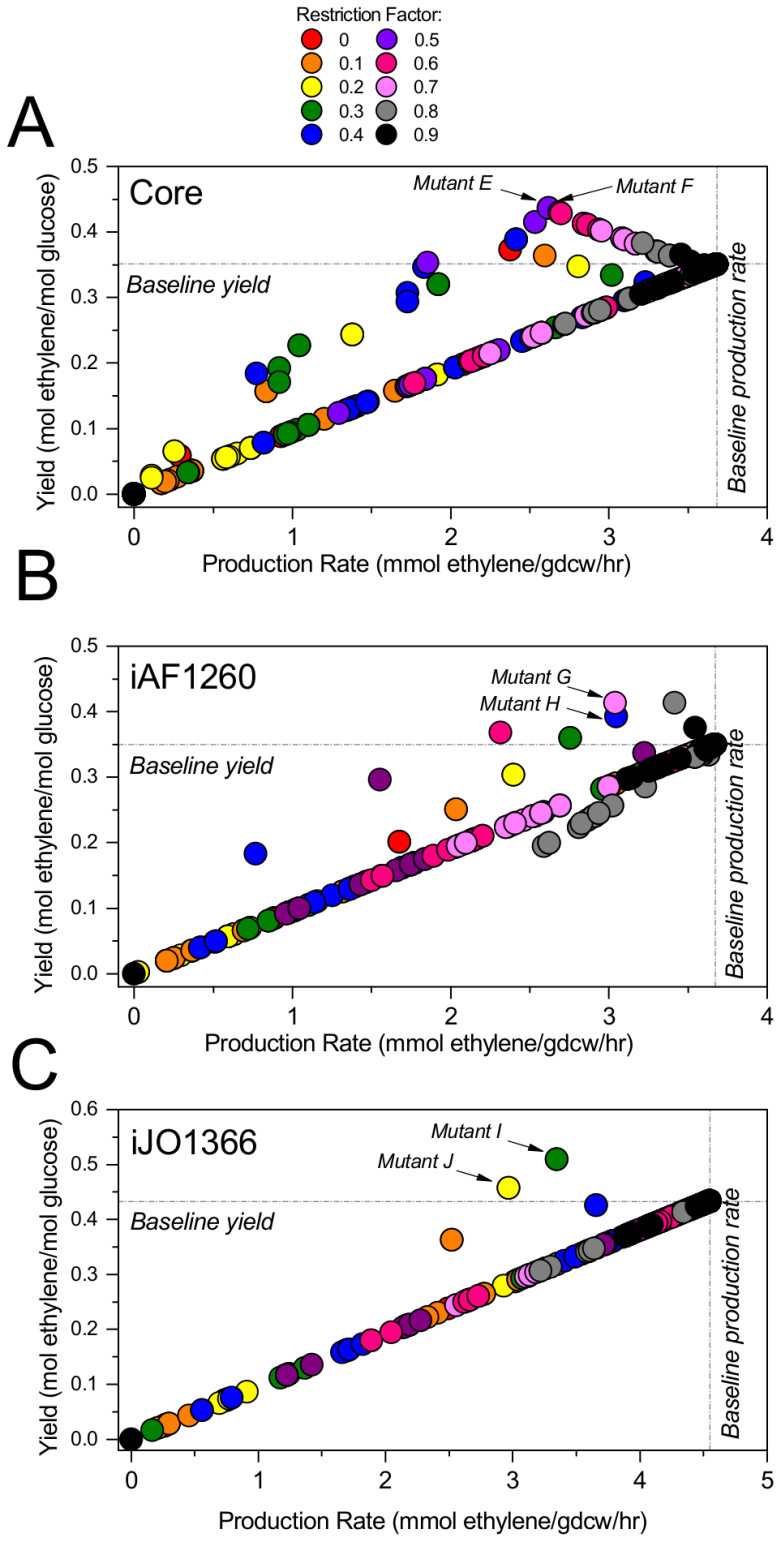
Tuning expression of individual reactions in the core model (A), the genome scale models iAF1260 (B) and iJO1366 (C). Constrictor was used to reduce each reaction individually by each of the restriction factors indicated in the legend, where each factor is a fraction of the baseline flux. Each point represents the ethylene yield and production rate of one Constrictor solution.

We analyzed the impact on ethylene yield when individual reactions were modified by a range of restriction factors, from complete knockout (f = 0) to slight reduction (f = 0.9). We maintained a minimum growth rate of 0.3 hr^−1^ and maximized ethylene demand. In the core model, numerous off-diagonal solutions are observed, many with yield increases (Fig. 3A). The highest yields in this study are obtained when baseline flux values are restricted by 50% (**mutants E** and **F**, Table 2). When enolase (ENO) or phosphoglycerate mutase (PGM) are targeted in mutant F, ethylene yield increases by 25% whereas when fructose-bisphosphate aldolase (FBA), phosphofructokinase (PFK), or triose-phosphate isomerase (TPI) are targeted in mutant F, yield increases by 23%. The mechanism for yield increase is similar to that for mutant A, wherein, ethanol production shuts off entirely when the flux requirement for NAD+/NADH balance decreases (solutions in Table S4 in File S1).

**Table 2:** Highest ethylene yielding strains obtained via Constrictor modification of individual reactions in three *E. coli* network models.

Strain	Reactions Modified (Individually)	Expression State	Ethylene Yield	% Δ Yield (with respect to baseline)	Growth Rate (hr^−1^)
***In core model:***					
Baseline	-	-	0.35	-	0.30
Mutant E	ENO, PGM	50% of baseline	0.44	+25%	0.30
Mutant F	FBA, PFK, TPI	50% of baseline	0.43	+23%	0.30
***In iAF1260:***					
Baseline	-	-	0.35	-	0.30
Mutant G	GLCtex	70% of baseline	0.41	+18%	0.30
Mutant H	GAPD, PGK	40% of baseline	0.39	+12%	0.30
***In iJO1366:***					
Baseline	-	-	0.43	-	0.30
Mutant I	GAPD, PGK	30% of baseline	0.51	+18%	0.30
Mutant J	GAPD, PGK	20% of baseline	0.46	+5%	0.30

The analysis was repeated using two genome scale models: iAF1260 (Fig. 3B), from which the core model was derived, and iJO1366 (Fig. 3C), the most current *E. coli* genome scale model. The baseline solution for each is obtained via the maximization of ethylene demand with constraints as described in Methods section. The baseline yield and production rate are found to be identical for the core and iAF1260 models (Table 2), though there are 437 reactions active in the iAF1260 baseline solution (Table S5 in File S1) versus the 72 active in the core baseline. The baseline solution in the iJO1366 model predicts a yield that is higher than that of the iAF1260 based models, at 0.43 mol ethylene/mol glucose, and includes 569 active reactions (Table S6 in File S1). When Constrictor is used to apply restrictions from 0 to 90% of the iAF1260 or iJO1366 baseline flux, higher yield and off-diagonal solutions are obtained. Neither the amount of flux restriction nor the targeted reactions that lead to the highest yield are completely consistent across models, though all top-performing targets impact glycolysis. The highest yield found in the iAF1260 model (18% increase over baseline, **mutant G**) is obtained when glucose transport is restricted to 70% of the baseline value, while the second highest yield (12% increase over baseline, **mutant H**) is obtained when GAPD or PGK are held at 40% of their baseline flux. As in mutant B and mutant H, the highest yielding solutions **mutants I** and **J** associated with the iJO1366 model are obtained by targeting GAPD or PGK. In mutant I, these reactions are constricted to 30% of baseline to provide an 18% increase in yield, and in mutant J, held at 20% of baseline for a 5% yield increase. For each of these mutants, ethanol production decreases by 100% with respect to baseline, indicating that oxygen limitation plays a role in suppressing the baseline yield. The complete flux solutions for each mutant are available in Tables S5 and S6 in File S1.

### Constricting functional classes of reactions reveals expression states that positively and negatively impact ethylene yield

One challenge facing all combinatorial approaches to study metabolic network behavior is the sheer size of the space that can be explored. In the core network, simulating every combination of major and minor restriction across every reaction would result in 10^53^ scenarios, many of which would be redundant. To reduce the combinatorial space when simultaneously modifying multiple reactions, we applied the Constrictor framework to restrict sets of metabolic reactions in various functional classes. The metabolic network is divided into fourteen functional classes, including glycolysis, the TCA cycle, pyruvate metabolism, anaplerotic reactions, oxidative phosphorylation, and ethylene production, among others (refer to Table S2 in File S1 for complete listing of reactions in each class). Sets of four to nine reactions were modified simultaneously in the core and genome scale networks, resulting in hundreds to thousands of expression states per study. The results of this assessment (using major and minor modifications of 20% and 80% baseline, respectively) in several functional classes are shown in Table 3. As found through individual reaction modification, a set of the expression states targeting glycolysis increase ethylene yield in the core model and in the model iJO1366, while restrictions to other subsystems decrease yield or create unsolvable bottlenecks. For most subsystems, the results were found to be consistent across models (e.g. for L-arginine biosynthesis, Exchange, TCA cycle), except for oxidative phosphorylation, in which case the core model was more heavily impacted (86% decrease in yields for some expression states) than either of the genome scale models. In iAF1260, none of the subsystems predicted yield increases for 20% and 80% restrictions, which is consistent with the results in Fig. 3, where the highest yielding solutions were obtained by directly restricting glucose exchange (GLCtex) to 70% or 80% of baseline.

**Table 3:** Maximum and minimum ethylene yield changes resulting from Constrictor modification of functional classes (f_minor_ = 0.8, f_major_ = 0.2).

		Core Model		iAF1260		iJO1366	
Functional Class	Reactions Modified (Simultaneously)	Max % ΔYield	Min % ΔYield	Max % ΔYield	Min % ΔYield	Max % ΔYield	Min % ΔYield
**Amino acid + Glutamate Metabolism + Anaplerotic reactions**	ASPTA, P5CD, GLNS, GLUDy, PPC	−26%	−83%	Not viable	Not viable	Not viable	Not viable
**L-arginine biosynthesis**	ACGK, ACGS, ACODA, ACOTA, AGPR, ARGSL, ARGSS, CBPS, OCBT	−20%	−80%	−21%	−84%	−21%	−83%
**Exchange**	EX_ac(e), EX_co2(e), EX_for(e), EX_etoh(e), EX_h2o(e)	−25%	−99%	−25%	−62%	−27%	−95%
**Glycolysis**	ENO, FBA, GAPD, PFK, PGI, PGK, PGM, PYK, TPI	+6%	−92%	−3%	−45%	+5%	−26%
**Oxidative Phosphorylation**	ADK1, ATPS4r, CYBD, PPKr, SUCDi, THD2, FRD7, NADH16	−21%	−86%	−0%	−2%	−1%	−6%
**Pentose Phosphate Pathway**	RPE, TALA, TKT1, TKT2	0%	−0.1%	−0.1%	−1%	0%	−0.2%
**Pyruvate Metabolism**	ACALD, ACKr, ALCD2x, PFL, PTAr	−2%	−8%	−1%	−4%	−2%	−8%
**TCA Cycle**	ACONTa, ACONTb, CS, FUM, ICDHyr, MDH	−21%	−85%	−21%	−83%	−21%	−84%

**Figure 4:**
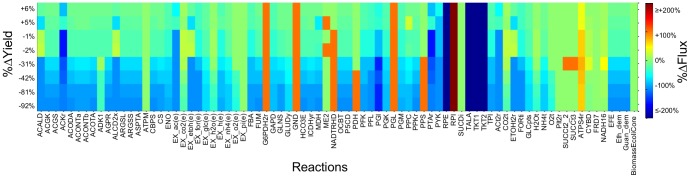
*In silico* modifications predicted by Constrictor targeting glycolysis. Eight unique flux vectors were obtained within the 65,536 expression states generated by simultaneously targeting ENO, FBA, GAPD, PFK, PGI, PGK, PGM, PYK, and TPI. These flux vectors are arranged (top to bottom rows) from highest to lowest percent change in ethylene yield relative to baseline yield. Reactions active in at least one expression state are arranged across the horizontal axis. Colors in the heat map depict the magnitude of the percent change in flux through each reaction (relative to baseline). Reactions that have zero flux for all combinations are not shown.

Restricting these subsystems does not reveal any expression states with yields higher than those found through individual reaction modification, but simultaneously modifying multiple reactions can provide a descriptive profile of the network. Targeting nine reactions in glycolysis (Table 3) gives rise to 65,536 expression states, which generate eight unique flux vectors in the core model (Fig. 4). By arranging the unique solutions in the form of a heatmap, key network characteristics are highlighted. We observe that, as yield decreases (top to bottom rows) the percent change in flux tends to decrease for most reactions. However, as the reactions that are not specifically targeted are allowed to vary between the original unlimited bounds, certain reactions experience increases in flux in response to Constrictor modifications. Reactions for which flux increases include G6PDH2r, GND, RPI, and PGL in the pentose phosphate pathway, which increase by a similar percentage for all expression states, suggesting that flux will divert through the pentose phosphate pathway for any restriction in glycolysis. The reactions that differ significantly between high and low yields are of particular interest. Flux through ATPS4r increases by a lower percentage in the solutions with improved yield than it does for the solutions with decreased yield: the lower yield solutions produce more ATP. This observation, combined with the fact that acetate exchange (EX_ac(e)) and acetate kinase (ACKr) also decrease more in the lower yield solutions, points to the importance of ATP/ADP balance. In this network, PFK, ACKr, and ATPS4r produce ATP, while the arginine biosynthesis pathway consumes ATP. When glycolysis is restricted, less carbon is available for processing by pyruvate metabolism, and acetate production decreases. This further limits ATP production, causing ATP synthase to balance the ADP produced by the arginine biosynthesis pathway. Thus, ATPS4r flux increases slightly with minor restrictions in glycolysis and continues to increase with major restrictions.

### Maximizing growth and applying Constrictor leads to export of ethylene and other products

All previous studies maximized ethylene production under simulated constraints. However, Constrictor is able to function on any baseline state. To demonstrate this potential, we maximized growth (via the biomass reaction) in the core, iAF1260, and iJO1366 models, then applied Constrictor with 20% and 80% restrictions on individual reactions to search for modifications that increase ethylene yield. None of the baseline solutions produce ethylene when growth is the objective (Table 4), which is not unexpected, as there is no native motivation for *E. coli* to divert resources towards ethylene biosynthesis. The baseline core achieves a maximum growth rate of 0.79 hr^−1^, while the genome scale models iAF160 and iJO1366 grow faster (0.85 hr^−1^ and 0.92 hr^−1^ respectively), likely due to differences in metabolites involved in the biomass reaction. When Constrictor is applied to the core model, no solutions result in ethylene production; however, expression states that produce ethylene are found when Constrictor is applied to iAF1260 and iJO1366 genome scale models. In either of the genome scale models, restricting L-glutamate 5-semialdehyde dehydratase reaction (G5SADs) results in ethylene production. Ethylene production is higher when G5SADs is modified by the major restriction factor (**mutants K** and **L** in Table 4) than when modified by the minor factor, though ethylene production is seen for either degree of restriction. G5SADs is a spontaneous reaction that is part of the proline biosynthesis pathway, which converts L-glutamate to L-proline. Limiting flux through this pathway increases flux through the L-arginine biosynthesis pathway (see ARGSL in mutant K in Table S5 in File S1 and mutant L in Table S6 in File S1). As L-arginine is a reactant in the EFE reaction, restrictions in G5SADs allow ethylene to be produced when biomass is the objective. Interestingly, targeting the two upstream reactions in L-proline biosynthesis (GLU5k and G5SD) or the downstream reaction (P5CR) does not cause ethylene to be produced. Restricting the other reactions in the pathway actually leads to decreases in flux through L-arginine synthesis. The differences between restricting the spontaneous G5SADs and the enzymatic reactions GLU5k, G5SDs, and P5CR may be due to the cofactor requirements of the enzymatic reactions. GLU5k is ATP/ADP dependent, while G5SDs and P5CR are NADP+/NADPH dependent. As described in previous sections, cofactor balances are crucial for achieving maximum ethylene yields.

**Table 4:** Strains producing ethylene when growth is the objective.

Strain	Reactions Modified (Individually)	Expression State	Ethylene Yield	Ethylene Production Rate	Growth Rate (hr^−1^)
***In core model:***					
Baseline	-	-	0	0	0.79
***In iAF1260:***					
Baseline	-	-	0	0	0.85
Mutant K	G5SADs	Major (20% of baseline)	0.03	0.29	0.82
***In iJO1366:***					
Baseline	-	-	0	0	0.92
Mutant L	G5SADs	Major	0.03	0.30	0.89

**Figure 5:**
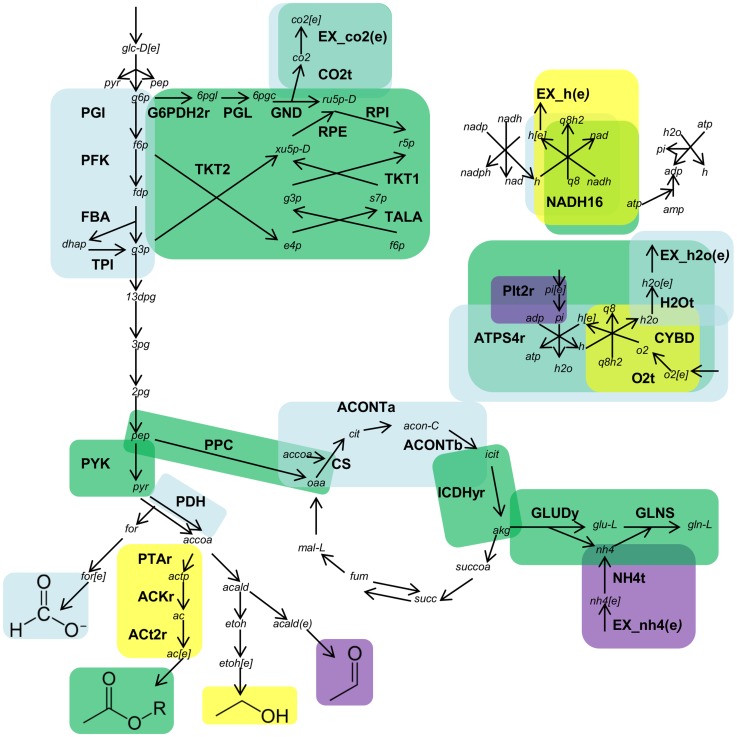
Constrictor identified overproduction of alternate metabolites when growth is the objective. Expression states that lead to increased formate, acetate, ethanol, or acetaldehyde production are located via Constrictor modification of each individual reaction in the core network (f_minor_ = 0.8, f_major_ = 0.2). Target reactions are highlighted by color, where blue reactions increase formate, green reactions increase acetate, yellow reactions increase ethanol, and violet reactions increase acetaldehyde yield.

We also maximize growth to demonstrate an alternate application of Constrictor; rather than looking for modifications that improve yield of a specific product, it also possible to scan a network to search *de novo* for desirable phenotypes. We applied Constrictor with f_minor_ = 0.8, and f_major_ = 0.2 to individual reactions in the core and genome scale model to locate modifications that increase production rate and yield of alternate metabolites. A summary of the targeted reactions in the core model that lead to increased production of ethanol, formate, acetate, or acetaldehyde is depicted in Fig. 5. In the core baseline state obtained from maximizing growth (Table 4), neither acetaldehyde, ethanol, nor formate is produced. Scanning with Constrictor reveals expression states that generate 0.41 mol ethanol/mol glucose, approximately 20% of the maximum theoretical yield on glucose [54]. The highest ethanol yields are obtained when either NADH16 or CYBD are restricted to 20% of the baseline value, which forces decreases in oxygen uptake and, as expected, shifts the metabolic network to utilize fermentation pathways. Similar increases in ethanol production are observed when oxygen transport is targeted in either the core network or genome scale networks, with the highest ethanol yield of 1.3 mol/mol glucose (65% of maximum theoretical yield) located in iAF1260. Select ethanol-producing solutions are included in Tables S4-S6 in File S1. Targeting oxygen uptake is expected to shift the network towards fermentation, but constricting many reactions within central metabolism can also increase fermentation product yield. In the TCA cycle for instance, targeting isocitrate dehydrogenase (ICDHyr) leads to increased acetate yield in the core model (Fig. 5) as well as in both genome scale models (Tables S5-S6 in File S1). As the genome scale models contain hundreds of exchange reactions (299 in iAF1260 and 312 in iJO1366), a number of potential products can be targeted. Several expression states in the Constrictor scan produce trace amounts of metabolites, such as D-lactate in iJO1366 when ICDHyr is targeted, and in iAF1260 with restrictions in Lipid A disaccaride synthase (LPADSS) or dihydroneopterin aldolase (DHNPA2). When ornithine decarboxylase (ORNDC) is limited, urea is produced; and with fumarase (FUM) limitations in iJO1366 or iAF1260, succinate is produced (Table S5-S6 in File S1).

## Discussion

Here, we present an FBA based optimization framework called Constrictor that predicts metabolic interventions by identifying unique “expression states” that give rise to overproduction of the biochemical target. We show that enforcing various levels of inhibition of flux constraints in metabolic network pathways can provide valuable engineering intervention strategies to achieve the overproduction target. We investigate heterologous ethylene production in *E. coli*, and demonstrate the capability of Constrictor to generate unique *in silico* mutants that are predicted to produce high ethylene yields. By restricting single enzymatic reactions or combinations of reactions, thousands of possible network states are revealed. In both reduced and genome scale models of *E. coli* metabolism, alternate expression states predict improvements in ethylene yield. By limiting individual reactions, disproportionate shifts in the network are forced, which reveal alternate solutions that highlight network characteristics. Constricting many reactions simultaneously provides a means to scan a larger portion of the solution space, which can be used to infer reactions or metabolites that have large impact on ethylene yield. In this analysis of ethylene production, we conclude that glucose and oxygen cannot be balanced at their maximum allowable values to achieve the highest ethylene yield. Thus anaerobic limitations result in ethanol production in the baseline networks. Constrictor suggests that the imbalance in the network can be remedied by reducing flux through glycolysis. Depending on the model, the highest ethylene yields are obtained for different target reactions and different restriction factor values, which emphasizes the importance of using a high throughput experimental approach, such as MAGE [14], to test a range of expression states in several potential target genes simultaneously. We also demonstrate the ability of Constrictor to search a network for targets that improve production of other metabolites. With biomass as the objective, we scanned and located major and minor expression states that increase export of formate, ethanol, acetate, or acetaldehyde. In genome scale models, additional products were detected in trace amounts by applying Constrictor to restrict individual reactions.

So far, improvements in ethylene production by selectively repressing metabolic activity have not yet been reported. Experimental attempts to engineer ethylene-producing microorganisms have included increasing media concentration, increasing gene copy number, inserting codon-optimized *efe* sequences, testing different promoters to drive *efe* expression, and reducing homologous recombination between *efe* promoter and chromosome [38], [40], [45], [55]. Flux balance analysis has not yet been applied to optimize ethylene production in *E. coli*. In the ethylene-producing networks described here, we focused on modifying only flux through the metabolic reactions initially present in the network, and did not attempt to substitute enzymes with different cofactors or force alternate substrates as has been suggested via computational analysis to optimize ethylene production in other organisms [26], [37]. Therefore, the mutants presented here could provide novel strategies to modify *E. coli* metabolism for ethylene production.

To achieve the overarching goal of creating robust platforms for predictive strain engineering, it is necessary to apply lessons learned from a multitude of FBA frameworks into experimental strain design strategies, including those that focus on improving computational efficiency [33], [34], [56], introducing novel and non-native pathways [57]–[59], defining biologically relevant objectives [60]–[62], incorporating experimental gene expression data [63], [64], and exploring gene addition [57], deletion, and over- or under-expression [29], [31], [57]. Unlike other FBA based optimization techniques, Constrictor provides a framework that samples an unexplored solution space by combinatorially analyzing various degrees of inhibitory effects of genetic mutations on the performance of biochemical networks. We demonstrate that by subtly or dramatically reducing flux through specific pathways, a distribution of solutions can be obtained that reveal novel metabolic engineering targets. Constrictor is a generally applicable technique for computationally generating and analyzing mutant strains, and can be incorporated into any metabolic flux analysis method that uses linear programming methodology, including regulated [64], [65], dynamic [24], and thermodynamic models [66], [67]. Because Constrictor operates independently of objective function, it has the potential to be used in conjunction with minimization of metabolic adjustment [60], regulatory on/off minimization [61], or with multi-objective optimization algorithms [68], [69].

Recent high-throughput recombineering techniques demonstrate that combinatorial exploration of the genomic space can reveal optimal mutants, while computational techniques prove to be well suited to analyze these combinations. Modifications suggested by Constrictor can be translated into *in vivo* modifications via a wealth of available strain engineering tools. Recently, characterization of promoter and upstream element sequences have enabled specific ranges of gene expression to be realized [70]–[72]. Similarly, emerging techniques that use small regulatory RNAs to target specific genes appear to be especially well-suited for implementing Constrictor predictions. The CRISPR interference system, which uses a catalytically inactive Cas9 protein to inhibit transcription of genes homologous to a guide RNA sequence, has been demonstrated to be tunable according to the location of the DNA target [73]. Implementing riboregulators is another strategy for achieving modular expression from selected genes [74]. In light of these and other advances in synthetic biology and genome engineering, it is becoming increasingly possible to achieve precise performance from biological parts, which allows for enhanced integration of strain engineering efforts and *in silico* predictions.

## Supporting Information

Figure S1 **Map of the core metabolic network for ethylene production.** The core metabolic network comprises of central metabolism, including glycolysis, the TCA cycle, and co-factor balancing equations (in grey box at top), as well as the arginine biosynthesis pathway. Ethylene production is carried out by the ethylene forming enzyme (EFE). Reaction abbreviations are in bold uppercase, and metabolite names are in lower case italics. Arrows indicate directionality of each reaction as allowed by the model. Refer to Table S2 in File S1 for the complete reactions used in the model, including the biomass reaction and list of all cofactors not illustrated in this figure.
(TIFF)

Figure S2 **Experimental growth rate of** ***E. coli*** **K12 MG1655.** *E. coli* (ATCC 700926) was grown at 37°C in M9 minimal media with 0.4% glucose. Optical density (OD_562_) was recorded every 20 minutes with a Tecan GENios plate reader (Tecan Group Ltd.) with Magellan software version 7.2. Normalized OD is presented (OD at time t divided by OD at t = 0). Error bars are standard deviation of n = 9 biological replicates. Growth rate (µ) was calculated with an exponential fit to the exponential growth portion of the curve (shadowed region in plot).
(TIFF)

Figure S3 **Trade-off between growth rate and ethylene yield with and without Constrictor.** The maximum theoretical ethylene yield was recorded for a range of minimum growth rates, with (f_minor_ = 0.8, f_major_ = 0.2) and without Constrictor. In each of the three models (labeled in panels), a similar trend is noted, with Constrictor yields approaching baseline yields as growth rate is increased.
(TIFF)

File S1 **Supplementary Tables.** **Table S1**: Reactions and Parameters. **Table S2**: Reactions in the core model for ethylene production from EFE. **Table S3**: List of metabolites in the core model. **Table S4**: Selected solutions from Constrictor in ethylene core model. **Table S5**: Selected solutions from Constrictor in genome scale model iAF1260. **Table S6**: Selected solutions from Constrictor in genome scale model iJO1366.
(XLSX)
