# Correction: CONSTRICTOR: Constraint Modification Provides Insight into Design of Biochemical Networks

**DOI:** 10.1371/journal.pone.0116651

**Published:** 2014-12-23

**Authors:** The PLOS ONE Staff

## Notice of Republication

This article was republished on December 11, 2014, to replace Equation 3, which was mistakenly left out of the web version of the article during the production process. The publisher apologizes for the error. Please view this article again to see the correct version.
