# Supplementary figures and images for: CONSTRICTOR: Constraint Modification Provides Insight into Design of Biochemical Networks
Source: PLoS One. 2014 Nov 25;9(11):e113820. doi: 10.1371/journal.pone.0113820 (PMC4244162; doi:10.1371/journal.pone.0113820)

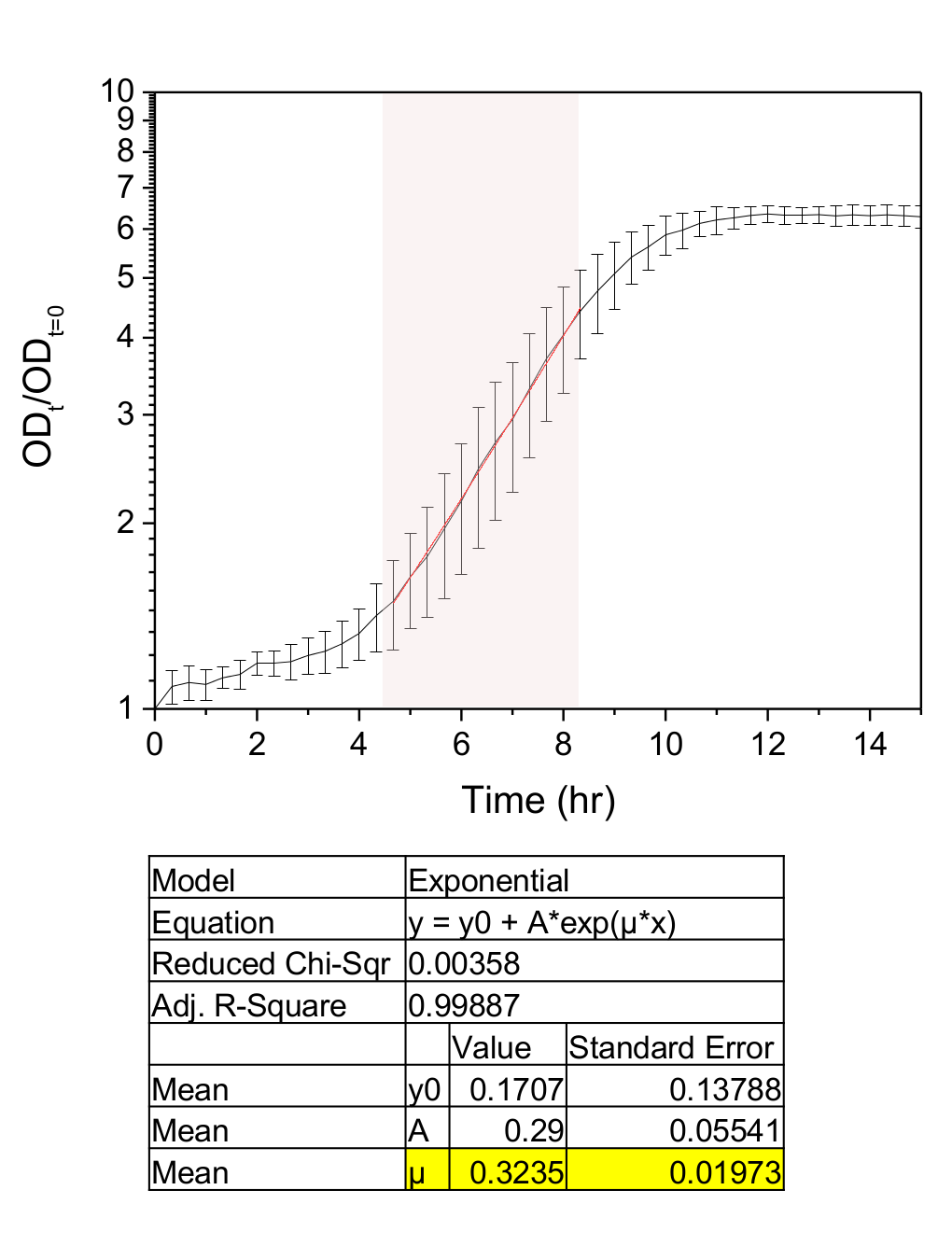

Supplement: Figure S2 — Experimental growth rate of E. coli K12 MG1655. E. coli (ATCC 700926) was grown at 37°C in M9 minimal media with 0.4% glucose. Optical density (OD562) was recorded every 20 minutes with a Tecan GENios plate reader (Tecan Group Ltd.) with Magellan software version 7.2. Normalized OD is presented (OD at time t divided by OD at t = 0). Error bars are standard deviation of n = 9 biological replicates. Growth rate (µ) was calculated with an exponential fit to the exponential growth portion of the curve (shadowed region in plot). (TIFF) [file pone.0113820.s002.tiff]

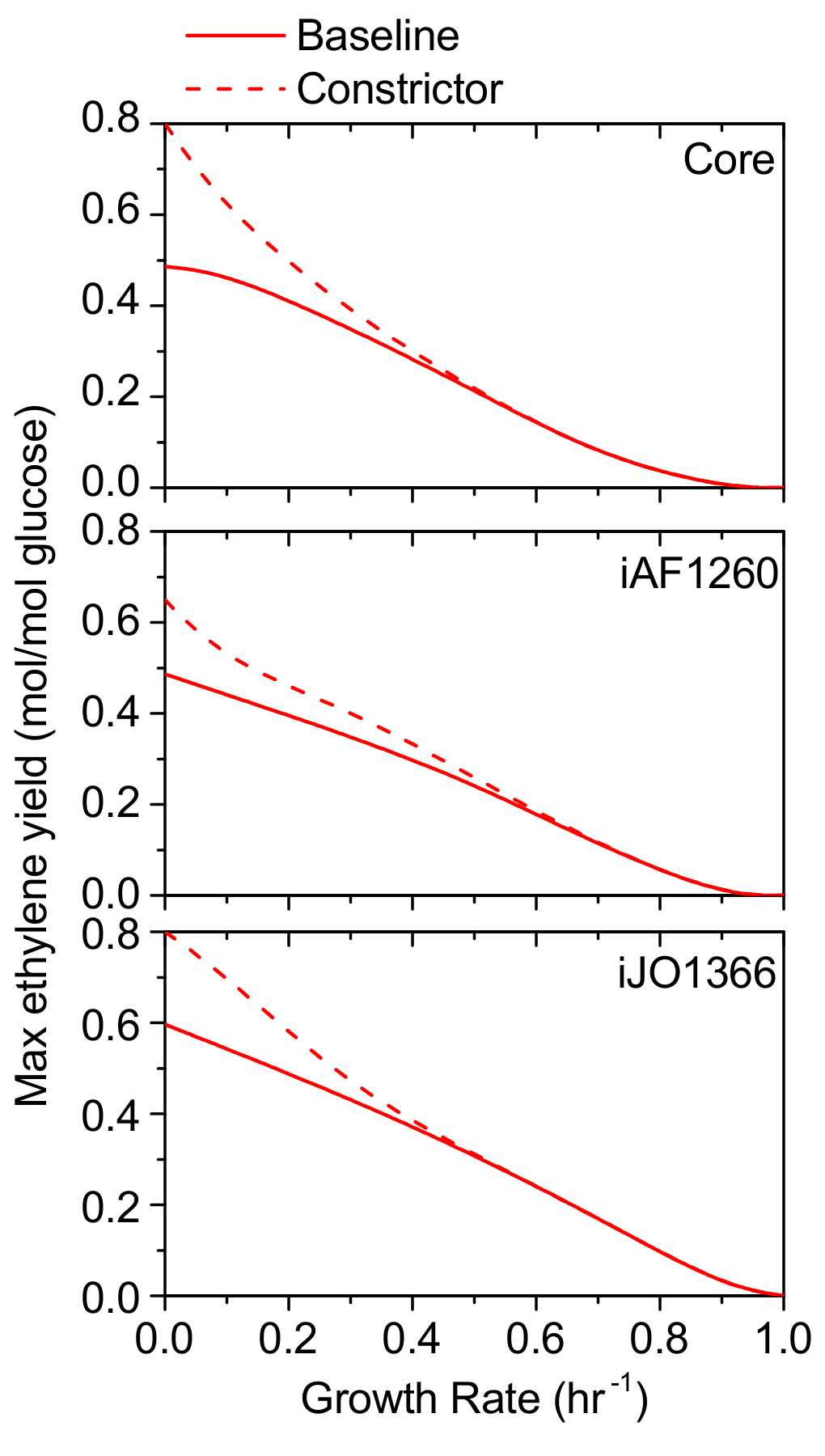

Supplement: Figure S3 — Trade-off between growth rate and ethylene yield with and without Constrictor. The maximum theoretical ethylene yield was recorded for a range of minimum growth rates, with (fminor = 0.8, fmajor = 0.2) and without Constrictor. In each of the three models (labeled in panels), a similar trend is noted, with Constrictor yields approaching baseline yields as growth rate is increased. (TIFF) [file pone.0113820.s003.tiff]
